# Lumbar transversus abdominis plane block: the role of local anesthetic volume and concentration—a pilot, prospective, randomized, controlled trial

**DOI:** 10.1186/s40814-015-0002-6

**Published:** 2015-03-25

**Authors:** Mauricio Forero, Andrew Heikkila, James E Paul, Ji Cheng, Lehana Thabane

**Affiliations:** 1 Department of Anesthesia, St. Joseph’s Healthcare Hamilton, McMaster University, 50 Charlton Avenue E, Hamilton, L8N 4A6 Ontario Canada; 2 Department of Anesthesia, MUMC-2V9, McMaster University, 1280 Main Street West, Hamilton, L8S 4 K1 Ontario Canada; 3 Department of Anesthesia, MUMC-2V9, Hamilton Health Sciences Corporation, McMaster University, 1280 Main Street West, Hamilton, L8S 4 L1 Ontario Canada; 4 Biostatistics/FORSC, St. Joseph’s Healthcare Hamilton, McMaster University, Martha Wing - 3rd Floor, H325, 50 Charlton Ave. E., Hamilton, L8N 4A6 Ontario Canada; 5 Biostatistics/FORSC, Clinical Epidemiology and Biostatistics, St. Joseph’s Healthcare Hamilton, McMaster University, Martha Wing - 3rd Floor, H325, 50 Charlton Ave. E., Hamilton, L8N 4A6 Ontario Canada

**Keywords:** Regional anesthesia, Transversus abdominis plane block, Perioperative pain management, Dermatomal blockade

## Abstract

**Background:**

The lumbar transversus abdominis plane (TAP) block has become an optional part of multimodal analgesia following several abdominal surgeries. There remains a lack of consensus regarding the extent of dermatomal blockade following lumber TAP block, as well as the optimal local anesthetic volumes and concentrations. The objectives of this pilot trial were to assess the feasibility of conducting a similar full-scale trial and gather information on relevant clinical outcomes, namely whether greater local anesthetic volumes would lead to more cephalad dermatomal blockade.

**Methods:**

The study was a prospective, double-blinded pilot randomized controlled trial (RCT) with three arms, each representing different local anesthetic volumes: 20 ml 0.5% ropivacaine, 30 ml 0.33% ropivacaine, and 40 ml 0.25% ropivacaine. We planned to recruit 30 females undergoing total abdominal hysterectomy for non-malignant pathology, who would then receive bilateral ultrasound-guided midaxillary TAP blocks at the completion of surgery. Randomized patients would be followed for 48 h post-block and would receive multimodal analgesia. The primary outcomes were measurements of patient recruitment and safety, to inform the feasibility of a larger trial. The main secondary outcome was the clinically pertinent endpoint of dermatomal blockade, which was assessed by loss of sensation to ice and pinprick.

**Results:**

Our target sample size was reached in 8 months, and the recruitment rate was 52% (31/60). A total of 58 TAP blocks were performed among 29 patients. All but one of the patients who received interventions were successfully followed and assessed up to 48 h. No patient safety-related adverse events were reported during the study period. The mean highest dermatome blocked in each group at any time point was T8. The 20 ml 0.5% ropivacaine group achieved a T9–L1 block that lasted for 48 h. The 30 ml 0.33% ropivacaine group had a sensory block from T9–L1 that regressed to T10–T12 between 24 and 48 h. The 40 ml 0.25% ropivacaine group reported an initial sensory block from T9–T12 that regressed by 24 h to include only the T12 dermatome.

**Conclusions:**

This pilot study demonstrated that the study design is feasible and safe to be carried to a full-scale RCT. The preliminary clinical findings showed that increasing the volume, while maintaining a constant dose, of local anesthetic does not appear to extend the height of dermatomal blockade following midaxillary TAP block. This finding needs to be confirmed in future studies.

**Trial registration:**

ClinicalTrials.gov registration is: NCT01307215.

## Background

An important component of pain after abdominal surgery is derived from the incision itself. The lumbar transversus abdominis plane (TAP) block has become a tool for the multimodal perioperative pain management for several surgical procedures, including total abdominal hysterectomy [1].

The initial technique was described by Rafi [2] in 2001 as a blind technique using surface landmarks at the Petit triangle and was further developed and tested by McDonnell [3-5]. Soon after this, an ultrasound-guided approach was introduced by Hebbard [6].

The aim of the block is to deposit local anesthetic in the plane between the transversus abdominis and internal oblique muscles and block spinal nerves T6–L1, which innervate the abdominal wall. However, the degree of sensory blockade has not been assessed in randomized trials and assessed inconsistently in observational studies [5-7]. A commonly identified deficiency in the literature is the lack of consensus regarding the optimal procedure-specific volumes and local anesthetic concentrations for lumbar TAP blocks. It is not yet clear whether the ultrasound-guided TAP block, as described by Hebbard [6], is sufficient for surgical procedures located at both the supra- and the infraumbilical levels, or whether upper abdominal procedures need an additional subcostal TAP block [8].

The primary objective of this study was to assess the feasibility of conducting a full-scale trial of similar design. Feasibility was measured as 1) whether the target patients could be successfully recruited and followed to the end of the study and 2) whether the designed interventions were safe. A secondary objective was to gather basic clinical and statistical information on the differences in TAP block height to inform a sample size calculation for a full study. The clinical objective for this study was to determine the dermatomal sensory block distribution in adult female patients undergoing total abdominal hysterectomy following the injection of three different local anesthetic volumes used with ultrasound-guided bilateral midaxillary TAP blocks. We hypothesized that greater local anesthetic volumes (i.e., 30 and 40 ml) would lead to more cephalad dermatomal blockade.

## Methods

### Design and eligibility criteria

This pilot trial was a prospective, randomized controlled, double-blinded study. The study was approved by the St. Joseph’s Healthcare Hamilton Research Ethics Board, and written informed consent was obtained from all subjects. Patients undergoing total abdominal hysterectomy via Pfannenstiel incision for non-malignant pathology, between the ages of 18 and 70 years with no history of chronic opioid use or abdominal surgery, were included in the study. Patients with a history of coagulopathy, local or systemic infection, local anesthetic allergy, or body mass index greater than 35 kg/m^2^ were excluded.

### Randomization

Subjects were randomized to one of three lumbar TAP block local anesthetic groups. Group A received 20 ml of 0.5% ropivacaine per side, group B 30 ml of 0.33% ropivacaine per side, and group C 40 ml of 0.25% ropivacaine per side. Randomization was achieved with 1:1:1 allocation ratio and took place intraoperatively with concealment by our hospital pharmacy until study completion. The patients, anesthesiologists, and post-block assessor were blinded to group allocation.

### Treatment procedures

All subjects underwent a standardized general anesthetic. Induction was performed using 2 mcg/kg of fentanyl, 1–2 mg/kg of propofol, and 0.6 mg/kg of rocuronium. Maintenance was achieved with sevoflurane at 1 minimal alveolar concentration (MAC), rocuronium as needed to keep two twitches using a nerve stimulator, and fentanyl 1 mcg/kg to keep systolic blood pressure within 20% of baseline. Thirty minutes before the end of surgery, morphine 3 mg and ondansetron 4 mg were administered to each patient.

Upon surgical completion, bilateral ultrasound-guided lumbar TAP blocks were performed while the patient was still under anesthesia and intubated. Three anesthesiologists, each skilled in lumbar TAP blockade, performed all blocks. Under sterile technique, a 12-MHz linear US transducer (GE Logiq E, Wisconsin, USA) was placed in a transverse orientation between the inferior costal margin and iliac crest in the midaxillary line. An 80 mm, 22-gauge EchoStim® needle (Benlan Inc., Oakville, ON, Canada) was advanced using an in-plane approach from medial to lateral and placed between the internal oblique and transversus abdominis muscles. Under real-time visualization, the randomized volume of ropivacaine was slowly injected followed by appropriate plane hydro-dissection.

The syringes containing the pre-mixed local anesthetics were placed in opaque containers by the hospital pharmacy and delivered to the operating room during the procedure. An anesthesia assistant aided in each TAP block and was responsible for confirming negative aspiration prior to injection of the local anesthetic. In this way, the anesthesiologist remained blinded to group allocation. The identical procedure was then performed on the contralateral side. The patient was extubated and transferred to the post-anesthetic care unit (PACU). All patients received a multimodal approach to pain management, including patient-controlled analgesia (PCA) opioid (morphine 1 mg IV q7min or hydromorphone 0.2 mg IV q7min), regular acetaminophen (975 mg PO q6h for 48 h), and ketorolac (10 mg IV q6h for 48 h). No neuromodulators or long-acting opioids were prescribed.

Perioperative data collection was performed by a research assistant not involved with patient care. Patients were assessed at 2, 6, 12, 24, and 48 h post-block. Numerous outcome measures, including sensory blockade, PCA opioid use, pain scores at rest and with knee flexion, incidence of nausea and vomiting, and sedation scores were assessed at each time interval. Overall patient satisfaction and time to PACU discharge were assessed by chart review at 48 h.

### Outcomes

The primary outcomes of this study were feasibility measurements. The feasibility of carrying a similar study design to a full-scale RCT was assessed in three respects: recruitment, follow-up, and safety. The success of recruitment was measured as eligibility rate (the number of eligible patients over the number of screened patients), recruitment rate (the number of recruited patients over the number of eligible patients), and the length of recruitment. The success of patient follow-up was reported as follow-up rate (the number of patients followed to the end of study period over the number of patients who underwent the intervention) and full data collection rate (the number of patients with all clinical endpoints collected over the number of patients who underwent the intervention). Patient safety was monitored by the incidence of pre-defined clinical adverse events (local anesthetic toxicity, bleeding, visceral injury).

Beyond these feasibility measurements, clinical information was collected as secondary outcomes for the purposes of gathering essential clinical and statistical information for future studies. Dermatomal block distribution, the primary clinical focus, was evaluated by loss of sensation to ice and pin prick. This was performed with a standard dermatomal map drawn onto each patient’s abdomen at the first post-block assessment. Each dermatome was assessed at four locations (right midclavicular, right sternal, left sternal, and left midclavicular), moving from T6 down to L1. Sensory change was compared to sensation at the C3/C4 dermatomes, with any reduced sensation counted as a blocked dermatome. Block failure rate was defined as the lack of any sensory block at all-time points following the TAP block.

Patient pain scores at rest and with knee flexion were assessed using the visual analog scale, with patients indicating their pain level on a continuous line between “no pain” (0) and “the worst pain of my life” (10). Postoperative opioid use was assessed by converting all opioids to intravenous morphine equivalents. Hydromorphone was converted to morphine by multiplying by a factor of five. Postoperative nausea and vomiting (PONV) was assessed using a four-point scale. A score of 0 indicated no nausea or vomiting, 1 mild (no treatment required), 2 moderate (treatment required and effective), and 3 severe (treatment required but not effective). Overall patient satisfaction was assessed by a five-point Likert score at 48 h. Time to PACU discharge was determined by chart review at 48 h.

### Sample size and statistical methods

As this pilot trial was designed mainly for the purposes of feasibility assessment, no formal sample size calculation was performed: the decision on size was primarily based on constraints of time and financial burden. The primary feasibility objectives were to successfully implement the study design while recruiting a target sample of 30 patients over a 6–8-month period and achieving more than 90% follow-up at 48 h. The feasibility outcomes were reported descriptively and narratively. For the clinical endpoints, only descriptive statistics, mean (standard deviation) for continuous outcomes and raw count (%) for categorical outcomes, were reported. Due to the nature of pilot designs, we chose not to conduct any informative statistical tests on the collected data. Statistical analyses were performed using Stata 10.2 (StataCorp LP, College Station, TX).

## Results

**Figure 1:**
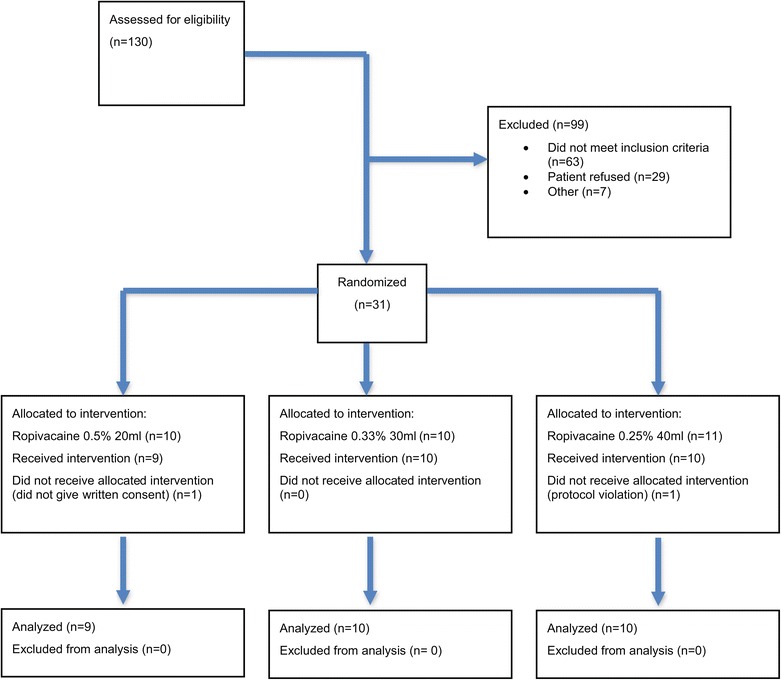
Consort flow study diagram.

The primary outcome of this pilot study was feasibility assessment including recruitment, follow-up, and patient safety. The details of the recruitment and follow-up of the study are shown in Figure 1. Recruitment occurred between March and November, 2011. One hundred thirty patients were initially screened. The eligibility rate was 46% (60/130). Among the 60 eligible patients, we obtained initial consent from 31 (recruitment rate: 52%) patients who were then randomized into three intervention arms. Among the 31 randomized patients, it was noted that one patient had failed to give signed consent, despite giving verbal consent, and one patient was found to have had protocol violation: these patients were removed from the intervention and their clinical data was not collected thereafter. A total of 58 TAP blocks were performed. Eighteen patients completed the full 48-h postoperative assessment. Among the patients who were not available for 48-h assessment, ten were discharged from hospital before 48 h and therefore were not considered as lost to follow-up: one patient who refused assessment at 48 h was considered as lost to follow-up (lost to follow-up rate, 3%). The time used to complete recruitment was 8 months. Regarding patient safety, no clinical adverse events were reported.

Baseline patient characteristics for the three intervention groups are reported in Table 1.

**Table 1:** Demographic characteristics of patients in the different treatment groups

Patient characteristics	20 ml 0.5% ropivacaine (*n* = 9)	30 ml 0.33% ropivacaine (*n* = 10)	40 ml 0.25% ropivacaine (*n* = 10)
Age (years)	43.1 (5.5)	44.1 (8.5)	50.0 (7.1)
Weight (kg)	73.7 (16.5)	64.6 (8.9)	75 (13.1)
Height (cm)	160.3 (6.7)	160.6 (5.2)	166.5 (7.9)
BMI (kg/m^2^)	28.5 (5)	24.7 (3.7)	27.2 (4.8)

Data presented as mean (SD).

**Figure 2:**
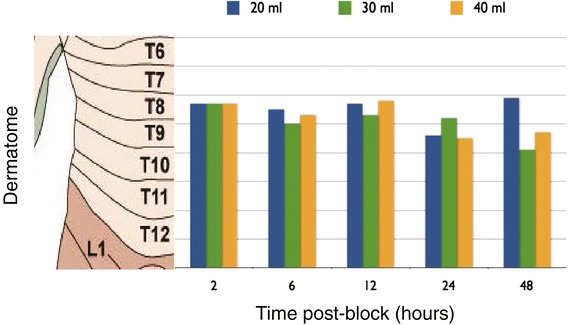
Mean maximal dermatomal blockade among the three groups over time.

**Figure 3:**
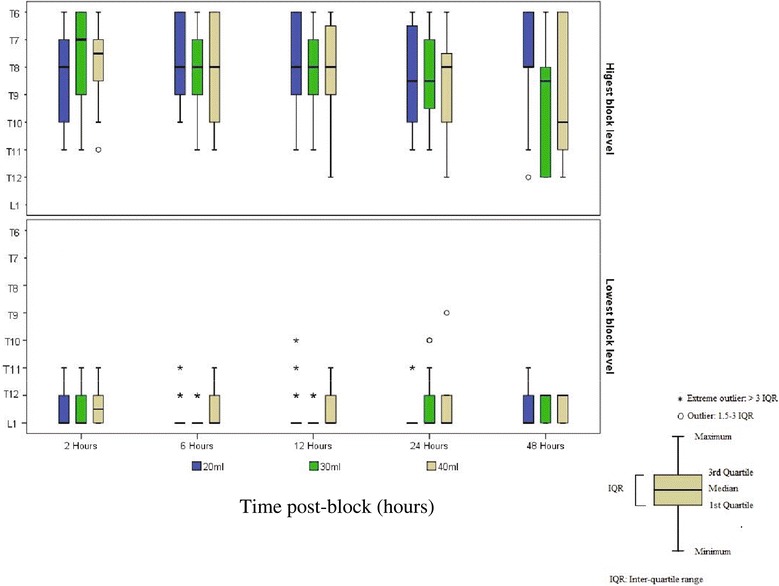
Median highest and lowest blocked dermatomes between the three groups.

There were no block failures in any patient. The mean highest dermatome blocked in each group at any time point was T8 (Figure 2). The mean maximal dermatome block was similar between 2 and 12 h. However, by 48 h, the highest mean sensory block dropped nearly one dermatomal level among all blocks. Figure 3 illustrates the distribution of the median highest and lowest blocks over time.

**Figure 4:**
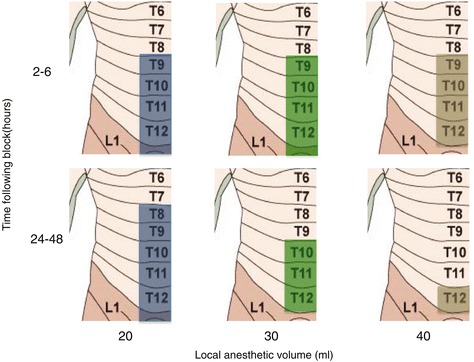
Dermatomal block behavior over time as a function of local anesthetic volume.

Figure 4 shows the change in sensory block at the sternum for each group, comparing “early block” (2–6 h) to “late block” (24–48 h). The presence of a dermatomal sensory block in more than 80% of the patients was considered significant. In group A, a sensory block between T9 and L1 was seen at each of the five time points. In group B, a T9–L1 sensory block was initially present but regressed to T10–T12 beyond 24 h. In group C, a T9–T12 block was achieved during the first 12 h, but only T12 blockade beyond 24 h. A T6 block was achieved unpredictably among the three groups less than one third of the time.

Midclavicular sensory block was less prominent than sternal blockade in groups A and B, with the same dermatome being blocked approximately 20% less often in the former. In contrast, group C had a similar percentage of dermatomes blocked in both locations. The behavior of the midclavicular blocks mirrored their sternal counterparts over time in all groups. A mean maximal block above T9 was not attained at the midclavicular level at any time point in any group.

The time to PACU discharge, along with other secondary clinical outcomes, is summarized in Table 2. There was a 50-min longer PACU stay in group B compared to group A (49.76, 4.72 to 94.8). Patient satisfaction was similar between groups. Total postoperative opioid use in group A was slightly higher. Group B used 26 mg less morphine than group A (−26.1, −75.9 to 23.8), while group C used 23 mg less than group A (−22.7, −74.72 to 29.34). With regard to pain scores at rest and with knee flexion, no meaningful difference was observed between the groups. At rest, groups B and C experienced less pain than group A (−0.4, −1.79 to 1, and −0.67, −2.01 to 0.79, respectively). However, in all three groups, less pain appears to have been experienced between 6 and 48 h when compared to 2 h (−2.5, −3.77 to −1.22). No clinical difference existed between nausea scores at any time point. Three patients in group A, one in group B, and two in group C reported severe nausea at one time point during assessment. Likewise, the sedation levels were similar.

**Table 2:** Comparison of secondary outcome measures of patients in the three treatment groups

Outcome measure	20 ml 0.5% ropivacaine (*n* = 9)	30 ml 0.33% ropivacaine (*n* = 10)	40 ml 0.25% ropivacaine (*n* = 10)
Total PCA morphine use (mg)	65.9 (63)	39.8 (25.2)	43.2 (36.9)
Time in PACU (min)	138.4 (38)	188.2 (54)	154.6 (43)
Satisfaction score	4.3 (3.5)	2.6 (2.5)	1.8 (1.3)
Pain score at rest (at 2 h)	6.1 (2.4)	5.3 (2.4)	5.4 (2.3)
Pain score with knee flexion (at 2 h)	6.7 (2.1)	5.0 (2.0)	4.9 (2.2)
PONV (at 2 h)^a^	6 (66.7)	3 (30.0)	3 (30.0)
Sedation (at 2 h)^a^	6 (66.7)	8 (80.0)	7 (70.0)

Data presented as mean (SD) for continuous and count (%) for dichotomous variables. ^a^Categorical outcome.

## Discussion

We enrolled total abdominal hysterectomy patients into a pilot RCT that assessed the feasibility of a trial that used three volumes of local anesthetic to achieve different block heights with a TAP block. We achieved our feasibility targets in that we recruited 31 patients over an 8-month period. Our recruitment rate was reasonably high and the loss to follow-up rate was quite low. It seems that patients are willing to give consent to participate and complete this type of trial. Further, with no serious clinical adverse events observed and only one patient dropping out of the study before completion for unveiled reasons, we believe that it is safe to conduct future RCTs under similar interventions. However, given our center had a mean recruitment rate of 3.9 patients per month, a definitive trial would take over 7 years at our center. To complete a definitive trial, a multicentre trial would be necessary such that it could be completed in a reasonable period of time. One potential setback was that of patients being discharged prior to completing the 48-h study period. In designing a future study, perhaps the study period can be reduced to the average length of hospital stay among the studied patient population.

The preliminary clinical finding of this study is the lack of volume effect in the cephalad spread of the midaxillary lumbar TAP block. Higher volumes of equal local anesthetic dose did not result in higher blockade nor did they translate to better analgesia outcomes or less opioid side effects. The strength of our study is that it was randomized and blinded and the follow-up was mostly complete. One could argue that our findings are limited by the fact that our methods varied both volume and concentration of local anesthetic, with the smallest volume group receiving the highest concentration of solution and the largest volume group receiving the least concentrated solution. Hence, the lack of volume effect could have been partially due to the effect of variable local anesthetic concentration. Other limitations of this study include the lack of weight-based local anesthetic dosing, a small sample size, and a BMI cutoff of 35 kg/m^2^, which limits generalizability.

Few studies have explored block distribution following TAP block. An observational trial by Lee et al. [9] assessed block distribution after the injection of 20 ml 0.5% ropivacaine either by posterior or subcostal approaches. The posterior approach led to T10–T12 blockade, while the subcostal to T9–T11 blockade. The highest dermatomes reached for the posterior and subcostal TAP blocks were T10 and T8, respectively. Our findings indicate that T9 and L1 are blocked more frequently than reported by Lee [9] and by Carney et al. [10] MRI contrast study, particularly in group A. A cadaver study by Rozen et al. [11] showed that nerves in the anterior axillary line have variable segmental origin from T9–L1, and perhaps midaxillary injection in our study allowed for more frequent blockade. Another cadaver study by Tran et al. [12] failed to show T9 staining, possibly due to postmortem contraction of fascial planes. Paravertebral spread may also play a role in achieving midthoracic blockade. Carney et al. [10] showed that contrast spreads to thoracic paravertebral spaces even after midaxillary injection.

Taken together, our results suggest that the 20 ml group achieved a consistent and lasting block that involved T9 to L1 dermatomes. Higher volumes of more dilute local anesthetic failed to result in longer lasting cephalad spread and were more prone to miss L1. In all groups, spread to T6 was achieved in a small number of patients, but consistent high thoracic blockade cannot be relied upon following a midaxillary block.

All three groups experienced some sensory blockade at 48 h, a seemingly long time after a single-shot block. While 48-h dermatomal blockade has not been assessed in previous studies, Carney et al. [13] showed superior analgesia at 48 h following TAP block with ropivacaine versus placebo block for total abdominal hysterectomies. It has been proposed that such prolonged analgesic benefit may be related to the poor vascularity of the TAP and therefore delayed local anesthetic uptake [13]. Several of our patients were discharged prior to 48 h and it is not known if there is additional dermatomal blockade beyond this time point.

With regards to other secondary outcomes, despite the presence of dermatomal sensory block, morphine consumption in all groups was higher than that reported in previous landmark-based TAP studies [3,4,13], particularly in group A (65.9 ± 63 mg). Carney et al. [13] found 48 h morphine consumption to be 27 ± 20 mg following TAP block. It is possible that this difference is due to differences in block technique. A recent systematic review revealed a trend towards prolonged analgesia following TAP block more posteriorly in the triangle of Petit [14]. A block in the triangle of Petit may block branches of T6–L1 nerves before they anastomose. Unfortunately, the above landmark-based studies which had reduced opioid consumption did not perform sensory block assessments, and it is therefore unknown if reduced opioid consumption was the product of clinically better blockade. TAP blocks address somatic pain, leaving the visceral component of pain intact. Griffiths et al. failed to show an analgesic benefit or opioid-sparing effect with TAP blocks for gynecological cancer surgery, possibly due to more extensive intraoperative visceral manipulation [15]. In our study, despite having somatic blockade, visceral pain likely contributed to significant morphine requirements. Assessing the nature of the patient’s pain would help to answer this question.

## Conclusions

In conclusion, this pilot study demonstrates that the study design is suitable and safe to be carried to a full-scale RCT but would likely require a multicentre approach. The preliminary clinical results showed that increasing the volume, while maintaining a constant dose of local anesthetic, does not appear to significantly extend the height of dermatomal blockade following single-shot midaxillary TAP block. The commonly utilized volume of 20 ml of more concentrated local anesthetic (in this case 0.5% ropivacaine) per side does appear to produce a consistent and long lasting low thoracic/high lumbar blockade. However, this finding needs to be confirmed in larger studies. Researchers interested in joining a future multicentre trial should contact the authors.

## Abbreviations

MAC: Minimal alveolar concentration
PACU: Post-anesthetic care unit
PCA: Patient controlled analgesia
PO: Latin “per os” meaning by mouth
PONV: Post-operative nausea vomiting
TAP: Transversus abdominis plane
